# Demonstrating cost-benefit for forensic laboratory resources: Project Resolution

**DOI:** 10.1016/j.fsisyn.2021.100158

**Published:** 2021-07-01

**Authors:** Ray A. Wickenheiser

**Affiliations:** New York State Police Crime Laboratory System, 1200 Washington Avenue, Building 30, Albany, NY, 12226-3000, USA

**Keywords:** Forensic laboratory, Cost-benefit, Resources, Case study, Backlog, Project Resolution, Recidivism, No-suspect sexual assault

## Abstract

The mission of forensic laboratories is to maximize the value of evidence. As price and quality are relatively fixed for consumers of forensic analysis, the main measure of service effectiveness is timeliness. Resources have been demonstrated as the means to improve timeliness. However, as the availability of governmental resources is also relatively fixed, to optimize resource distribution, there must be an evaluation as to the most effective allocation. Cost-benefit analysis or determination of the return on investment has been demonstrated as an objective means to compare various competing options as targets for resource deployment. Therefore, a cost-benefit analysis of forensic science will be conducted to evaluate the option of applying additional resources to maximize the value of forensic analysis through optimal timeliness of service. A case study will be provided using historical data to examine the net benefit from forensic investigative leads.

## 1 Background

To those outside of the forensic laboratory enterprise, it is potentially puzzling as to why one case can be completed in one week, while another may take a year from the day it is was submitted to when a report is received. The actual analysis of a case is frequently much quicker than the typical forensic laboratory response time. The culprit may be a backlog, which lengthens response times.

**Table 1:** Project resolution return on investment.

	Cost of Sexual Assault	CODIS Hits	Project Resolution Cost	Recidivism Factor	Return on Investment
Conservative Model	$111,238	164	$286,000	7	$446.51
Aggressive Model	$435,419	164	$286,000	26.22	$6546.63

Although no official definition exists for the DNA backlog, the National Institute of Justice (NIJ) defines a backlogged case as one that remains untested for 30 days after it has been submitted to a laboratory.^1^ Rather than arbitrarily assign a 30-day post submission period, a forensic laboratory total backlog can be defined as all of the cases submitted to the forensic laboratory that are not yet completed. While simplistic, this definition does not permit insight into what comprises the total backlog. Therefore, to better describe and understand what comprises the total backlog of cases submitted but not yet completed, breaking this larger total backlog into two more defined backlogs is useful. These two more descriptive backlogs are the backlog of cases in-analysis, or where the case is actively being analyzed, and the backlog of cases that are awaiting analysis to begin [1,2].

It has been hypothesized in reimaging forensic laboratory service that the ideal response time would include elimination of the backlog of cases awaiting analysis [1]. With no backlog of cases awaiting analysis, case analysis would commence immediately, thereby increasing the value of evidence by providing the timeliest response possible. The timeliest response is the time actually taken by analysis, rather than including the addition of time spent awaiting analysis to begin. While this approach sounds simple, obtaining the resources to generate sufficient supply of analysis to meet or exceed demand has proven elusive. The vast majority of forensic laboratories are governmental, and even non-governmental vendor labs rely on government provided resources for payment for analysis conducted to support investigations. With a relatively fixed pie of taxation and other revenues available to governments, an increase of resources to one sector comes with intense scrutiny as it frequently occurs at the expense of another. Therefore, allocation of scarce governmental resources typically includes an analysis of cost and benefit, to determine the optimal resource allocation. While directing investigations to the correct suspect via an investigative lead earlier in the investigation appears to be very sound reasoning, providing a cost-benefit analysis would further objectively support or refute this hypothesis.

To evaluate the net benefit from forensic science, a study was conducted using data from no-suspect sexual assaults. If forensic service is lacking, investigators will resort to traditional non-forensic investigative techniques to solve cases, therefore using no-suspect cases is a means to evaluate cases that are reliant on forensic DNA databases to generate leads [3]. Without those leads and an eyewitness to identify a suspect, no-suspect sexual assault cases are quite dependent on forensic analysis for resolution. Further, a slow response time could lead to investigator triage of cases and samples and also increase the potential for pursuing the incorrect suspect or a wrongful conviction. Therefore, no-suspect cases represent an excellent model for cost-benefit analysis, as they are seldom solved without forensic intervention and represent an elevated risk for adverse outcomes.

## 2 Project Resolution

Acadiana Criminalistics Laboratory (ACL) is a quasi-governmental crime laboratory located in New Iberia, Louisiana. ACL serves a population of approximately 600,000 in Southwest Louisiana, which is comprised of the 8 parishes (counties) of Acadiana including Acadia, Evangeline, Lafayette, Iberia, St. Landry, St. Martin, St. Mary, and Vermilion parishes. ACL is independently funded via court costs and other levies, operating under the guidance of an independent volunteer commission of law enforcement, justice system and elected government officials.

In the midst of the South Louisiana Serial Killer Case investigation, ACL scientists theorized the identity of the serial killer could lie within the many previously examined pre-DNA serological cuttings saved at the laboratory. The South Louisiana Serial Killer was abducting, sexually assaulting and murdering women in the Baton Rouge and Acadiana Region of Louisiana. His DNA profile was linked to semen located in six homicides occurring in 2001 and 2002, with no matching profile found in NDIS (National DNA Index System).

In the early days of serological examinations at ACL prior to the advent of forensic DNA technology, analysts routinely retained many sample cuttings from sexual assault cases at the laboratory, while the bulk of evidence was returned due to storage considerations. In a project named Project Resolution, hundreds of cases with these cuttings were reviewed, specifically searching for unsolved sexual assault cases with DNA potential. A team of laboratory staff combed through laboratory case files to marry the cuttings with case documentation, distilling case candidates to 605 cases of unsolved sexual assault where cuttings from potential semen stains were present. The Louisiana legislature was approached to fund the project. $186,000 was allocated in a special project to outsource these cases to a private DNA vendor, Orchid Cellmark Laboratories in Dallas, Texas.

The 605 case cuttings were analyzed for male DNA profiles originating from semen. 317 semen positive cases were identified from these 605 no suspect “Cold” cases dating back to 1985, which equates to 52.4% positive rate. 285 of these 317 cases, or 90%, produced a foreign male profile. As per CODIS (Combined DNA Index System) requirements, qualified ACL scientists technically reviewed the vendor cases and entered the forensic profiles into NDIS. Numerous DNA hits between forensic profiles and known offenders were realized almost immediately upon profile entry. The number of hits rose by the day as the database was being populated with convicted individuals.

As of April 6, 2011, in an update for Project Resolution outcomes, there were 134 CODIS matches in these cases to 119 offenders. This equals a 47% hit rate (134/285). Of these 134 matches, 19 cases or 14.1% were made to out of state offenders. A recent update from ACL Director Kevin Ardoin on March 9, 2021 indicated that over the last 10 years an additional 30 matches have occurred, totaling 164 matches out of 285 profiles [4]. This equates to a 58% hit rate to convicted offenders, arrestees, or to other cases. The CODIS hit rate increased from 47% to 58% in the last 10 years due to DNA database expansion, as no additional forensic (crime scene) profiles have been added.

These data convey a comprehensive history of previously unsolved cases from the Acadiana region served by ACL, demonstrating the historical impact of a forensic DNA program to solve sexual assaults. Several cases are detailed to highlight the predatory nature of the crimes and aspects of recidivism within that large study of sexual assault crimes. Early in the timeline of Project Resolution, at the time the CODIS hits were first occurring, laboratory staff assembled a white board to track cases. Cases and hits were color coded and grouped to show how different cases, offenders, and locations cross matched. Due to the size and complexity of the project and the volume of data available, the true scope of the information was difficult to visualize, there was so much investigative data realized.

## 3 Ville Platte serial rapist

Grouping cases through color coding and categorization by location enabled the discovery of several serial offenders, some of which are highlighted. One such perpetrator was operating the in the small Louisiana community of Ville Platte, committing five unsolved sexual assaults which were linked through an identical male DNA profile originating from semen at each crime scene. The unsolved sexual assaults had occurred between 1987 and July 2001. In four of the five assaults, the victims had also been beaten or choked, and in the fifth the perpetrator sexually assaulted the victim while 3 young girls were present in the residence.

Once investigators were alerted of the sexual assault trend, they examined their cases for potential links. The DA's investigator requested ACL DNA Technical Leader George Schiro to check the forensic DNA profile from a 1999 sexual assault against the unsolved string of sexual assaults [5]. A reference sample from the victim's boyfriend was submitted as a potential suspect. The subsequent comparison of DNA profiles had ruled out the boyfriend. In the examination of the mixed forensic profile, similarities were noticed between the survivor's contribution and that of the serial offender, indicating a potential relative of the survivor could be the assailant in the other unsolved cases.

The Ville Platte serial rapist case was solved by matching his sister's DNA from a forensic mixed profile and familial comparisons assisted by Dr. George Carmody's KinTest© program. Expertise gained using familial relationships evaluated with KinTest© Likelihood Ratios in the South Louisiana Serial Killer Case was used to evaluate the survivor component in relationship to the Ville Platte serial rapist DNA profile. The KinTest© software program utilizes the frequency data of the STR core loci to mathematically compare competing hypotheses of the samples being related versus unrelated. It provides a Likelihood Ratio (LR) of two people being related as full siblings, half siblings, cousins, or parent-child versus being unrelated. Fifteen out of 26 alleles from the 13 core CODIS loci in use at the time were in common between the survivor component and the serial rapist profile. Using KinTest© a likelihood ratio of 3200 was developed, meaning the serial rapist was 3200 times more likely to be a full sibling to the source of the reference sample versus an unrelated individual [5].

With this investigative information regarding the immediate family of the survivor, investigators determined she had 5 brothers. The first brother examined was in jail in Texas. A keyboard search of his profile eliminated him as the serial rapist. CODIS rules at the time dictated that sibling information could not be released. However, KinTest© also revealed a LR of several thousand in the analysis of the Ville Platte Serial rapist with the Texas brother, further indicating a full sibling relationship as quite likely.

A second brother, Norman Wilson, resided in Bossier City, Louisiana. When approached by investigators, he provided a voluntary DNA sample. His DNA profile matched the DNA profile of the rapist from the five unsolved cases. A sixth matching sexual assault was located that was not included in Project Resolution due to a filing oversight. Of the six victims, four were deceased while the fifth did not wish to pursue charges. Evidence of the pattern of sexual assaults was introduced in the case of aggravated sexual assault that was brought against Norman Wilson. He plead guilty to one count of forcible sexual assault and received a thirty-five-year sentence for his series of crimes [[5], [6], [7]].

In the South Louisiana Serial Killer case (Derrick Todd Lee) investigated in 2001 and 2002, a now expunged local DNA database was used to search for a potential family member to the forensic profile [8]. Potential family member candidates were sought for voluntary elimination samples. As male candidates were compared to the DNA profile from the homicides, LRs were also developed using the Carmody KinTest© software to determine closeness of their relationship with the forensic profile. After approximately 16 comparisons of potential relative profiles to the forensic profile to evaluate for closeness of relationship, the case eventually hit a dead end due to the presence of an adopted individual whose family lineage could not be traced to further related individuals. While reference profiles were obtained with full consent of these individuals, there was a desire not to delve too deeply into very personal specific situations such as the adoption. Once that situation was discovered, a decision was made not to pursue this line of investigation, which was unique at the time.

The South Louisiana Serial Killer Case was subsequently solved via a direct DNA hit to a suspect Derrick Todd Lee after a composite sketch was released, following a DNA inclusion to a trace profile obtained from sweat droplets deposited on the denim dress of an attempted sexual assault survivor. That experience permitted comparisons and objective evaluations of the victim's forensic mixture profile and the Ville Platte serial rapist profile to determine her full sibling was a likely suspect. That investigative information lead to the solving of six unsolved sexual assaults and proved the value of software to evaluate potential familial relationships.

## 4 Lafayette serial rapist

Among the many serial predators identified by Project Resolution were eight unsolved sexual attacks occurring between 1986 and 1998 in Lafayette, Louisiana [9]. Upon notification by ACL scientists that the unsolved crimes were connected to the same individual, Lafayette City Police investigators reopened the cases. In their review of the cold cases they discovered a fingerprint which had not been entered into the database. The print was linked to a known property criminal, who was subsequently called in via his ankle bracelet to provide a known DNA sample. The resulting profile hit upon the eight unsolved sexual assaults [9]. In the ensuing press conference called to announce the solving of the outstanding sexual offenses, when asked how the crimes were solved, the lead investigator responded, “good old-fashioned police work.” Clearly, forensic technology combined with investigative skills is a better mechanism to solve crime.

## 5 Ellen Crawford

Crime creates a lasting impact on victims. Rather than being defined by the crime, survivors have realized their stories empower them to speak with the voice of experience and legitimacy of few others. Their voice of experience can drive legislative action. Once case involved Ellen Crawford, also of Lafayette, Louisiana [10]. The survivor of a particularly brutal sexual assault and attempted murder, Ellen spoke out in favor of the expansion of CODIS in Louisiana to include DNA from arrestees. Ellen was stabbed multiple times, beaten, and abducted from the parking lot of a large retail store in broad daylight on a Sunday morning while picking up supplies for her church. She was driven to another location where she was subsequently sexually assaulted. After that assault, while the suspect drove to a more secluded area, Ellen was sure that she would become a homicide victim. Seeing farmers in a nearby field, she leapt from the speeding vehicle, being driven over as the suspect fled. While laying critically injured in the road as the farmers ran to her aid, she willed herself to live to prevent the crime to be perpetrated on future victims [10]. Ellen understood recidivism.

Officials called upon Ellen to draw upon her direct experience to formulate her opinion regarding upcoming CODIS expansion legislation aimed at expanding to arrestees. In that press conference in which local public officials were grilled regarding the proposed implementation of arrestee testing, the press conference was stoned silent for several minutes after Ellen told her story and advocated for a law which could prevent her crime befalling future victims. While law had been passed in Louisiana enabling the collection of DNA from arrestees in 1997, the lack of funding had prevented the DNA database from becoming operational, let alone test arrestees [11]. The new proposed law passed with a resounding majority, with only 2 votes against. As a result of the aftermath of the Derrick Todd Lee case and the advocacy of victims including Ellen Crawford, Louisiana was the first state to enact a DNA arrestee database and begin collection starting in October of 2002 [11].

## 6 Business case

Use of a business case provides financial estimates of cost and benefit to back up the reduction of pain and suffering and increased risk of wrongful suspicion and conviction [3,[12], [13], [14]]. The cost estimates apply the common metric of dollars to not only demonstrate the relatively high cost of sexual assault to survivors and society, dollars also permit cross comparisons between competing expenditures. Thus, an objective comparison can be permitted to provide justification for allocation of resources towards those enterprises exhibiting greater savings and impact due to specific spending.

The original cost of the vendor DNA laboratory for Project Resolution to generate DNA profiles from potential semen stains was $186,000, which yielded 285 CODIS profiles, from which 164 matches occurred. Cases and profiles were reviewed by ACL scientists first to obtain the potential samples and then to review case data and documentation and upload profiles to CODIS. An estimated 10 scientists worked one week to review the cases, prepare, and ship the samples, or 50 person days. A technical review of CODIS profile is estimated to take 4 hours (1/2 day), which amounts to approximately 142.5 days for review time. Combined, 192.5 person days, or approximately one year of scientist time was utilized to prepare the 285 cases and profiles for CODIS upload and comparison. Fully loaded scientist salaries were approximately $100,000 per person year at that time. Detailed estimates of forensic scientist compensation are available [15], however as the project was very dynamic, involving multiple sections, pay grades, salary schedules, staff juggling multiple duties, and the elapsing of time since the project, a general estimate of cost was provided to account for these variables. Therefore, the total cost of Project Resolution was approximately $286,000.

Estimates of the cost of sexual assault range from $111,238 [3] to $435,419 [14]. If a single sexual assault could have been prevented for each sexual assault that was solved in Project Resolution, then between $18,243,032 ($111,238 X 164 sexual assault cases) and $71,408,716 ($435,419 X 164 sexual assault cases) was the cost savings benefit. At a total cost of $286,000, this equates to between $63.79 and $249.68 cost savings benefit per dollar spent. It should be noted that the studies listed [3,14] deal with the residue of untested cases and not the immediate testing of new cases. This is an important distinction since the ROI from backlogged cases nets out those cases where the statute of limitations has run its course, however that would not be an issue with immediate testing of new cases.

These savings between $18.2 Million and $71.4 Million assumes saving only a single victim per CODIS hit, versus including a factor for recidivist behavior. This behavior was notably at play for the both the Ville Platte and Lafayette Serial Rapists. It is also noteworthy that many victims do not report their ordeal to the authorities [16]. A vast majority of sexual assault reports are truthfully reported, with only between 2.1 and 7.1% reported to be false claims, supporting testing of all sexual assault cases [15]. There are also many cases that despite a report of sexual assault, collection of a sexual assault kit, submission to the forensic laboratory and DNA analysis, frequently a CODIS profile is not obtained. Using the technology available at the time of Project Resolution, 285 of the 605 sexual assault case candidates yielded a CODIS profile, or 47.1% (285/605 × 100). Of those 317 cases which had semen identified, 32 or 10.1% did not generate a CODIS qualifying profile (32/317 × 100). Therefore, the 285 case yielding profiles are representative of a much larger underlying body of offenses. Many more sexual assaults likely occurred than those which were fortunate enough to have samples saved and generate CODIS eligible DNA profiles.

Recidivist factors have been calculated to determine how many sexual assaults are potentially prevented for each sexual assault which has been solved [3,14]. These factors range between 7 [3] and 26.22 additional sexual assaults [14]. When applied to the 164 CODIS hits generated by Project Resolution, the return on investment soars to $446.50 using the most conservative estimates to $6546.63 per dollar expended using the most aggressive estimates (see Table 1). Regardless of the level of conservatism, the return on investment for conducting DNA analysis and using resulting forensic profiles to search CODIS is compelling. This massively positive return on investment as demonstrated by savings of the cost of crime, backed by the actual case circumstances detailed through cases solved by Project Resolution provide economic support for resources necessary to conduct forensic analysis on cases of sexual assault.

## 7 Discussion

The concept of proportionality has been discussed as means to determine options, where each option has potential negative impacts [2]. Proportionality provides for a comparison of the positive and negative of the options to be weighed against each other. As a result, the greater good in imperfect options can be established where neither choice has all positive impacts. Policies can be established to lessen negative impacts of the chosen solution, acknowledging that there are potential harms that must be avoided. Hence, proportionality can be used to assess options where one choice may come at the expense of another, which is the case for allocation of limited resources. Options for resource allocation should be laid out with pros and cons, such that objective metrics and data provide economic support for the ethical and moral justification of the best option choice(s).

The Ville Platte and Lafayette serial rapists, the Ellen Crawford case and Project Resolution as a whole, demonstrate the value of conducting timely forensic analysis. Each case involved multiple victims preyed upon by serial predators, who could have been disrupted earlier in their path of crime to spare future victims. The lab director of ACL in 2003 was quoted by the local newspaper that 5 of 6 homicide victims would have been saved had a DNA databank been in place in Louisiana prior to the reign of terror perpetrated by Derrick Todd Lee. Lee had a committed a number of offenses that would have placed him in the DNA database, hence a timely analysis of the first case would have enabled a match to Lee, thereby with his apprehension, the remaining homicide victims would have been saved. Hindsight is 20:20. Now that one knows the power of a DNA database and timely processing, the case has been made to utilize these tools to their fullest crime solving and crime preventing extent.

Project Resolution offers a historical perspective of the potential of forensic investigation of sexual assault of the Acadiana Criminalistics Laboratory's 8 parish area of Acadiana. Serving a population of approximately 600,000, these data can be extrapolated to examine forensic potential in other jurisdictions, or used as a model to evaluate and demonstrate forensic potential. It has been said that an ounce of prevention is worth a pound of cure. While matching 164 cases in a single project demonstrates that crime solving potential of forensic DNA analysis coupled with NDIS to develop suspects, analysis came far too late in the criminal career of perpetrators to prevent victims. It does illustrate the large number of victims who could have been spared their trauma if forensic analysis and a well populated DNA database were in place.

Any time expended beyond the minimum time for forensic analysis represents unnecessary exposure of society to additional victimization. Forensic technology and DNA databases are now in place. What is left is to make the case to balance analytical supply with demand to make best use of these tools. Leaving the same perpetrators additional time to commit the same crimes on new victims flies in the face of this learning from past experience. Earlier resolution of cases not only leads to fewer victims, it increases chance of rehabilitation, yielding lesser sentences due to a lower level of crime perpetrated. The result is savings on incarceration, less investigative and court costs, and a reduced opportunity to question or convict the wrong individual. The time has come for reimagining forensic science through applying past lessons to our future investigative systems.

## 8 Conclusion

No-suspect sexual assaults represent a case type where forensic evidence is frequently present, and also where other investigative methods are often less effective. As a result, no-suspect sexual assaults can be used to demonstrate the net benefit or return on investment of applying objective forensic laboratory analysis to provide investigative leads. Project Resolution conducted in Acadiana Criminalistics Laboratory in 2002–2003 and subsequent database hits realized through CODIS provide a large concrete study of sexual assault cases to demonstrate the recidivist nature of crime, and the crime solving and potential crime preventing value of forensic science analytical capability. Conducting forensic analysis and database searching is extremely well supported by a business case, demonstrating savings between $446.51 and $6546.63 dollars saved for every dollar spent. This business case provides a model for evaluating support for forensic resources to conduct forensic analysis immediately, thereby maximizing the value of evidentiary value.

## Declaration of interests

The author declares that he has no known competing financial interests or personal relationships that could have appeared to influence the work reported in this paper.

## Footnotes

1 U.S. Department of Justice, Office of Justice Programs. (November 2011). The DNA Backlog. OJP Fact Sheet.
